# The Development of Muscle Fatigue Suppresses Auditory Sensory Gating (P50) during Sustained Contraction

**DOI:** 10.3389/fnsys.2016.00044

**Published:** 2016-05-20

**Authors:** Aleksander A. Aleksandrov, Elena S. Dmitrieva, Ludmila N. Stankevich, Veronika M. Knyazeva, Anna N. Shestakova

**Affiliations:** ^1^Department of Higher Nervous Activity and Psychophysiology, Saint Petersburg State UniversitySaint Petersburg, Russia; ^2^Centre for Cognition and Decision Making, National Research University Higher School of EconomicsMoscow, Russia

**Keywords:** sensory gating, auditory P50 suppression, schizophrenia, central muscle fatigue, event-related potentials, mismatch negativity, attention, preattentive auditory information processing

## Abstract

Our aim was to study the influence of fatigue development on sensory gating during a muscle load. The fatiguing task was sustained contraction of a handgrip dynamometer with 7 and 30% maximum voluntary contraction (MVC). The suppression of P50, an auditory event-related potential, was used as the sensory gating index in the paired-click paradigm with a 500 ms interstimulus interval; the difference between the P50 amplitudes of the first and the second stimuli of the pair was used as the sensory gating index. We found that the 30% MVC fatigue development strongly decreased sensory gating, sometimes totally suppressing it. We concluded that central fatigue impaired motor performance and strongly suppressed inhibitory processes, as shown by the decreased P50 amplitude to the second stimulus. Therefore, muscle central fatigue influences sensory gating, similar to schizophrenia spectrum disorders.

## Introduction

Sensory gating is an important mechanism for processing incoming information. This process helps the brain prevent an overload of irrelevant sensory information. The suppression of P50, which is an auditory component of the event-related potentials (ERPs) that occur in response to a pair of single-sound stimuli, is a common, noninvasive technique for studying sensory gating capability (Freedman et al., 1987; Braff and Geyer, 1990; Weisser et al., 2001).

Freedman et al. (1983); Freedman et al. (1987) were the first to focus on P50 (Freedman et al., 1983, 1987; Waldo and Freedman, 1986). Those researchers used a paradigm with two identical stimuli (S1 and S2) and a 500 ms fixed, interstimulus interval. When compared with the S1 P50 amplitude, the S2 P50 amplitude was significantly lower. The difference between the amplitudes of S2 and S1 became the index of suppression or the sensory gating index. This paradigm is still relevant for recent studies (Griskova-Bulanova et al., 2011).

Sensory gating is a preattentive process, which occurs involuntarily on a preconscious level. In a single-stimulus paradigm, the P50 amplitude is not influenced by attention (Hillyard et al., 1973; Hackley and Graham, 1987). In the paired-stimuli paradigm, S1 and S2 attentional manipulations also fail to influence P50 amplitude and suppression (Jerger et al., 1992; White and Yee, 1997).

It is possible that preattentive processes, such as sensory gating and mismatch negativity (MMN), have similar features. For example, schizophrenia spectrum disorders significantly disable the sensory gating mechanism and also reduce the MMN amplitude (Näätänen et al., 1978; Braff and Geyer, 1990; Todd et al., 2012). Gjini et al. (2010) showed that a positive correlation exists between sensory gating and MMN. The greater the sensory gating index was, the greater the MMN amplitude was in response to the abstract stimuli (the paired-stimuli oddball paradigm, which consists of frequent, standard ascending frequency tone pairs and rare descending frequency tone deviants, was used).

Recent data from our laboratory showed that the central fatigue that occurred during heavy muscle load significantly influenced MMN, reducing its amplitude (Evstigneeva et al., 2010). Thus, we supposed that fatigue development might also affect sensory gating mechanisms. In the present study, we examined the changes in P50 suppression that occur during muscle fatigue development.

## Materials and Methods

The standard paired-click paradigm was used. Stimuli were presented as paired, 1 ms broadband white noise clicks (84 dB) with a 500 ms interstimulus interval and a random 6–8 s interpair interval. Sounds were given binaurally via calibrated headphones. A background white noise (20 dB) was used to hide possible external sound interference. Stimuli were created and presented using the Psytask v.1.41.2 Software (Mitsar Co. Ltd., St. Petersburg, Russia Federation).

To provide a muscle load, we used sustained contraction of a handgrip dynamometer (KRG-4 T10, Nobel Elektronik, Karlskoga, Sweden) with 7% and 30% maximum voluntary contraction (MVC). The force of compression and the target level were measured using Force Feedback v. 2.0 (National Instruments Corporation, TX, Austin, USA). Before the experiment, the MVC was defined as the maximum dynamometer compression averaged over 5 s. The MVC was also measured after a 7% MVC load and after a 30% MVC load. This approach provided an objective measurement of fatigue. To quantify the subjective rating of perceived effort (RPE) during physical activity, the 10-point Borg scale was used (Borg, 1998).

The experiment consisted of four blocks, with 100 paired stimuli in each block:

- 1st block: “control”—passive listening task and no muscle load.
- 2nd block: “7%”—listening task with sustained 7% MVC contraction.
- 3rd block: “30%”—listening task with sustained 30% MVC contraction.
- 4th block: “recovery”—passive listening task and no muscle load.

The rest periods between the blocks were 2, 5, and 5 min, respectively.

The electroencephalogram (EEG) was recorded using a 21-channel, digital EEG amplifier (Mitsar-EEG-05/70–201: Mitsar Co. Ltd., St. Petersburg, Russia Federation; bandwidth 10–100 Hz, sampling frequency 500 Hz) and the WinEEG v. 2.4 Software package (Mitsar Co. Ltd., St. Petersburg, Russia Federation). The EEG data were collected from seven channels (F3, F4, C3, C4, Cz, P3, and P4) using silver-silver chloride electrodes according to the international 10–20 system (Jasper, 1958). References were placed on the earlobes, and the ground was placed on the forehead. To track eye movement artifacts, an electrooculogram was recorded. The electrode resistance did not exceed 5 kΩ.

**Figure 1:**
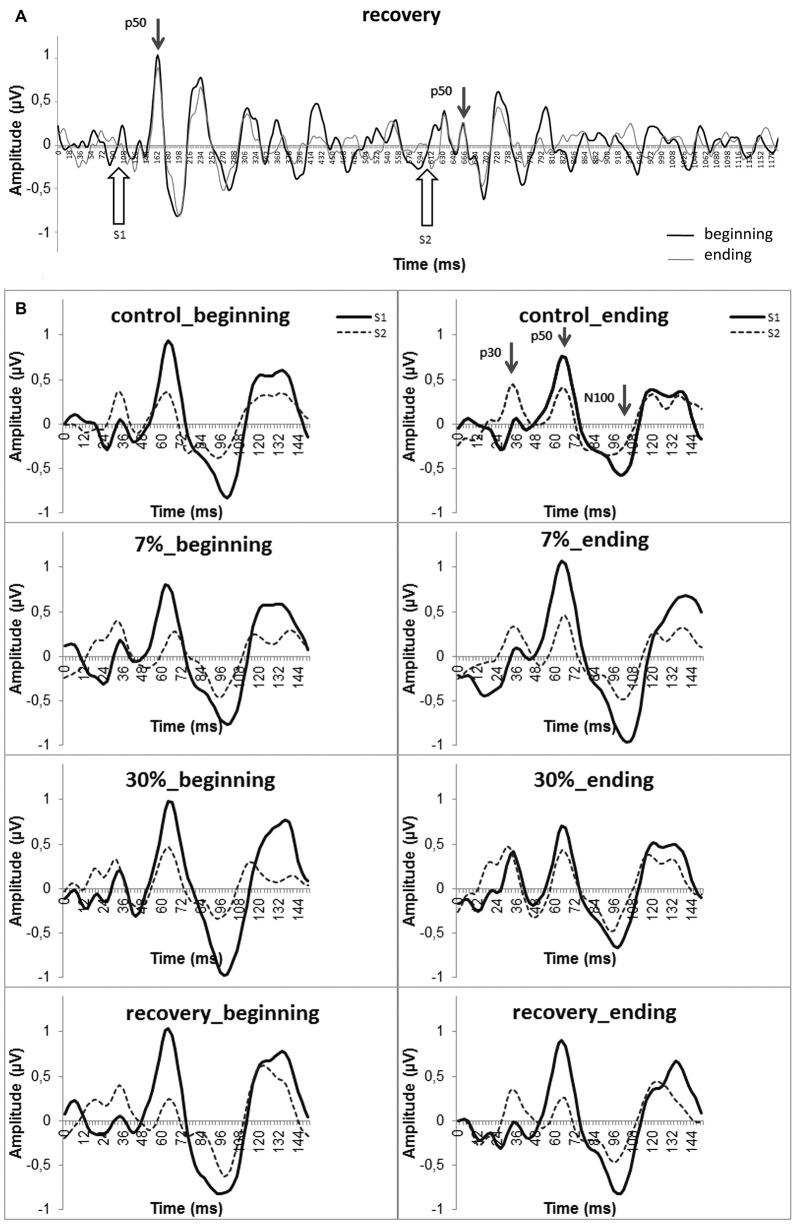
**Grand—averaged ERPs at the Cz electrode. (A)** Group average (*n* = 25) of the ERPs in the 4th block (i.e., recovery after physical load). The P50 amplitudes in response to the first and second stimuli are marked with arrows. Black line (“beginning”)—the first half of the experimental block; gray line (“ending”)—the second half of the experimental block. **(B)** Dynamic changes in P50 components for all blocks (“beginning” and “ending”). Smooth line—S1 ERP, dotted line—S2 ERP. Evoked potential components are marked by arrows in the upper right panel.

The bandwidth 10–100 Hz filter that was used is optimal for studying P50 (Rentzsch et al., 2008a, b; Figure 1). The EEG was filtered, and ERPs were created using WinEEG.

Twenty-five adults (8 men and 17 women), who ranged in age from 19 to 28 years (mean age = 22.9 years), participated in the experiment. All subjects were right-handed, mentally healthy, nondrug users, and nonsmokers, and none of the subjects had brain concussions. The subjects were asked not to drink tea or coffee less then 2 h before the experiment.

All subjects gave written, informed consent prior to the study. All experimental procedures were approved by the local ethics committee (Saint Petersburg State University, Russia), according to the Declaration of Helsinki.

P50 was measured as the maximum peak in the 40–80 ms range from the beginning of the stimulus. Amplitude was measured from the N40 to P50 peaks (Rentzsch et al., 2008a, b). The sensory gating index was measured by extracting the stimulus 2 amplitude (AS2) from the stimulus 1 amplitude (AS1). We could not measure the sensory gating index by division (AS1/AS2), as reported in some studies, because some subjects had fully suppressed P50 for stimulus 2 and the AS2 was zero.

To study the dynamic influence of fatigue on P50 suppression, each block was divided into two equal subgroups: the subgroup “beginning” (first 50 stimuli) and the subgroup “ending” (last 50 stimuli). The averaged ERP was measured for each subgroup. The “peak to peak” amplitudes diagram for P50 was presented.

The data were analyzed using two-way repeated measures ANOVA, with the factors of fatigue (control, 7%, 30%, and recovery) and dynamics (beginning, ending). In *post hoc* comparisons, Tukey tests were used, and *p* ≤ 0.05 was considered significant.

## Results

**Figure 2:**
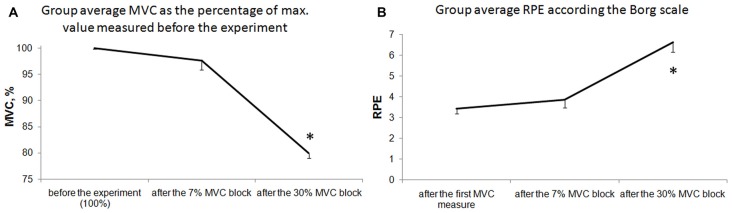
**Fatigue indexes**. Maximum voluntary contraction (MVC) decrease in % **(A)** and rating of perceived effort (RPE) according the Borg scale **(B)** before the experiment, after the 2nd block (7% MVC) and after the 3rd block (30% MVC). The bars indicated the standard error. Stars denote significant differences (*p* < 0.001).

We found that the 7% MVC load did not lead to pronounced muscle fatigue, but a significant degree of fatigue occurred during the 30% MVC load. This fatigue was demonstrated by a significant decrease in the MVC values and an increase in the RPE after the 3rd block (30% MVC load; Figure 2). No significant difference was observed between the MVC and RPE values measured before the 1st block and the values measured after the 2nd block (7% MVC load), but the difference between the values measured before the 1st block and after the 3rd block was significant (*p* ≤ 0.01); the RPE values increased, and the MVC decreased.

**Figure 3:**
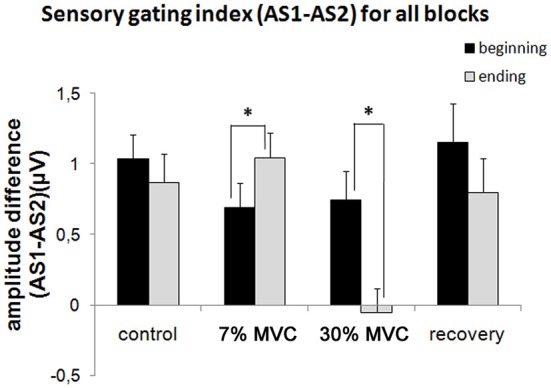
**Sensory gating indexes (AS1-AS2) for all experimental blocks**. Black columns (“beginning”)—the first half of the experimental block (responses to the first 50 stimuli); white columns (“ending”) — the second half of the experimental block (responses to the last 50 stimuli). The bars indicated the standard error. Stars denote significant differences (*p* < 0.05).

Figure 3 shows the sensory gating indexes for all experimental blocks. Two-way ANOVA revealed a significant main effect of fatigue (*F*_(3)_ = 5.69, *p* = 0.0015) and a significant fatigue by dynamics interaction (*F*_(3)_ = 4.99, *p* = 0.003). The sensory gating index (AS1-AS2) at the beginning and end of each block changed only with the muscle load (7% MVC and 30% MVC load blocks). A significant increase in the sensory gating index (*F*_(1)_ = 5.19, *p* = 0.03) occurred in the 7% MVC load block, and a strong decrease (*F*_(1)_ = 8.17, *p* = 0.009) also occurred in the 30% MVC load block.

No significant difference was observed between the sensory gating indexes measured at the beginning and at the end of the blocks with no muscle load (control and recovery blocks). Thus, no change in the sensory gating index occurred during the passive listening tasks.

**Figure 4:**
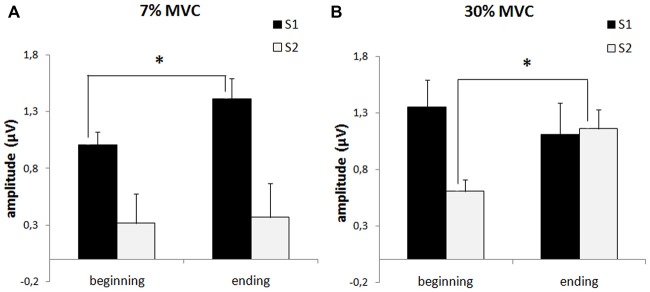
**P50 amplitude in response to the first stimulus (AS1, black columns) and to the second stimulus (AS2, white columns) during fatigue development (“beginning” and “ending”) in the 2nd (7% MVC) block (A) and the 3rd (30% MVC) block (B)**. The bars indicated the standard error. Stars denote significant differences (*p* < 0.05).

We analyzed which of the stimuli had the most influence on the sensory gating index change that occurred in the 7% and 30% MVC load blocks. The data showed (Figure 4) that during the strong muscle load (30% MVC), there was a minor decrease in the AS1 and a significant increase in the AS2 (*p* = 0.046). During the 7% MVC muscle load, there was a significant decrease in the AS1 (*p* = 0.006) and the AS2 did not change.

In summary, the sustained 30% MVC contraction caused strong muscle fatigue and significantly decreased the sensory gating index. Furthermore, a reversion occurred (i.e., in some subjects, AS2 became larger then AS1). The low muscle load (7% MVC) contributed to the sensory gating index (AS1-AS2) increase.

## Discussion

Our results demonstrate that the fatigue development that occurs with a 30% MVC strongly decreases sensory gating, sometimes completely suppressing sensory gating. The 2nd block (i.e., the listening task with a nonfatiguing sustained 7% MVC contraction) shows that the muscle load *per se* does not cause the sensory gating suppression. We believe that central fatigue impairs motor performance and also strongly decreases inhibitory processes; these effects are connected with a decrease of the P50 amplitude related to the second stimulus, leading to a strong reduction of sensory gating.

The increase of the sensory gating index (AS1-AS2) that occurred during the low force condition (7% MVC) supports the observation that some levels of muscle contractions may facilitate cognitive performance (Zijdewind et al., 2006). Although P50 is not a definite component of cognitive performance because it refers to preconscious processes, our data suggest that low muscle load may facilitate some preattentive processes.

It should be noted that the sensory gating recovery is rapid, and after the short 5-min pause, the difference between the AS1-AS2 amplitudes is normalized and becomes even greater than the difference observed in the control block (Figure 3).

Evstigneeva et al. (2010) studied the influence of fatigue on MMN and showed that precognitive brain processes are influenced by central fatigue mechanisms. Those authors found that strong fatigue decreases the MMN amplitude, but low muscle load leads to an insignificant increase in the MMN amplitude.

Another probable explanation for the AS1 increase and AS2 decrease that occurred in the 3rd block (30% MVC) is the influence of muscle pain after the strong muscle load. The influence of pain on P50 has only been studied in patients with chronic lower back pain (Fann et al., 2005), who have an increased P50 latency and a decreased amplitude, as well as a tendency toward reduced second stimulus P50 suppression. We suppose that muscle fatigue may influence preattentive auditory information processing. This effect may be caused by a decline in cognitive performance in patients with chronic fatigue (Busichio et al., 2004) that occurs during the muscle load task (Lorist et al., 2002; Zijdewind et al., 2006).

Central fatigue induced by a strong muscle load significantly decreases the sensory gating index and the MMN amplitude (Evstigneeva et al., 2010). Such fatigue also impairs cognitive task performance (Fleury and Bard, 1987; Lorist et al., 2002; Zijdewind et al., 2006). In the case of the deterioration of cognitive functions that is provoked by motor fatigue, the completion of a motor task during muscle fatigue development requires an increasing amount of attentional resources (Fleury and Bard, 1987; Lorist et al., 2002; Zijdewind et al., 2006). It is also possible that preattentional resources are used.

In the present study, we show that low muscle load (7% MVC) influences the P50 component in response to the first stimulus of the standard paired-stimuli paradigm. This fact is of special interest because in works that studied the influence of attention on P50 amplitude (Guterman et al., 1992), attention was found to influence only the amplitude of the response to the second stimulus of the pair (AS2). Low muscle load (7% MVC) increases the sensory gating index by increasing the response to the first stimulus (AS1) of the pair. High muscle load (30% MVC) decreases the sensory gating index by increasing the response to the second stimulus (AS2) of the pair.

In conclusion, the development of central fatigue development during muscle fatigue strongly reduced sensory gating, sometimes completely suppressing sensory gating. We identified a new factor that inhibits sensory gating in addition to the sensory gating suppression that was previously identified in schizophrenia. We propose that central fatigue impairs motor performance and also blocks the basic sensory gating inhibitory processes that underlie the decreased P50 amplitude in response to the second stimulus.

## Author Contributions

AAA provided the concept of the study. AAA and ESD provided design of the study. ESD, VMK, LNS and ANS contributed data acquisition and analysis. All authors contributed to the interpretation and discussion of the results, drafted and critically revised the manuscript.

## Funding

The work of AAA, ESD, LNS and VMK was supported by the Russian Science Foundation under Grant No. 14-25-00065. The work of ANS has been funded by the Russian Academic Excellence Project “5-100”.

## Conflict of Interest Statement

The authors declare that the research was conducted in the absence of any commercial or financial relationships that could be construed as a potential conflict of interest.

## Abbreviations

ERP: event-related potential
MMN: mismatch negativity
MVC: maximum voluntary contraction
RPE: rating of perceived effort.
